# Supplementary material for: Polyketide synthase gene clusters in the ericoid mycorrhizal fungus Oidiodendron maius: genome-wide prediction, expression profiling, and functional characterization of knockout mutants
Source: Front Plant Sci. 2026 Sep 16;17:1924430. doi: 10.3389/fpls.2026.1924430 (PMC13623903; doi:10.3389/fpls.2026.1924430)
Supplement: Supplementary file 1 [file SupplementaryFile1.zip › Supplementary Materials/Supplementary Folder 1.zip!/Oidma1_AssemblyScaffolds_Repeatmasked/knownclusterblast/cluster49/jgi.p_Oidma1_35127_mibig_hits.html]

| MIBiG Protein | Description | MIBiG Cluster | MiBiG Product | % ID | % Coverage | BLAST Score | E-value |
| --- | --- | --- | --- | --- | --- | --- | --- |
| AFT91387.1 | EcdD | BGC0000338 | Echinocandin\_B\_biosynthetic\_gene\_cluster | 45 | 91.3043478261 | 418 | 9.5e-117 |
| EAL89045.1 | MFS\_sugar\_permease,\_putative | BGC0000355 | Fumiquinazolines\_biosynthetic\_gene\_cluster | 29 | 86.4130434783 | 155 | 1.5e-37 |
| CDG12868.1 | putative\_drug\_efflux\_protein | BGC0001415 | Althiomycin\_biosynthetic\_gene\_cluster | 28 | 28.9855072464 | 50 | 8.9e-06 |
